# Resolving Challenges in HIV Cure–Related Research: Protocol for a Modified Delphi Consensus-Building Process

**DOI:** 10.2196/67123

**Published:** 2025-08-06

**Authors:** John Sauceda, Anastasia Korolkova, Lidia Rodriguez Garcia, Bridgette Picou, Ali Ahmed, Karine Dubé

**Affiliations:** 1 Division of Prevention Science University of California, San Francisco San Francisco, CA United States; 2 University of California, San Diego San Diego, CA United States; 3 The Well Project Brooklyn, NY United States

**Keywords:** hybrid Delphi, consensus building, HIV cure research, analytical treatment interruption, recruitment, underrepresented populations, protocol

## Abstract

**Background:**

HIV cure–related research is expanding rapidly, bringing both new opportunities and ethical challenges. Historically, clinical trials for novel HIV treatments have underrepresented populations most affected by HIV, such as Black gay men and transgender women. This disparity is compounded by medical mistrust and historical mistreatment of racially and ethnically diverse individuals in the United States. Addressing these issues is crucial as we plan HIV cure–related clinical trials. We aim to build consensus on how to increase representation of groups most affected by HIV in cure-related trials in the United States.

**Objective:**

We aimed to describe a protocol using a hybrid Delphi consensus-building methodology to build consensus on 3 key research questions: how to better engage populations in HIV cure research who carry the greatest burden of HIV (ie, racial, ethnic, sex, and gender minority groups); how to enhance trust and diminish mistrust in health care and scientific settings that influence willingness to participate; and how to design HIV cure research and analytical treatment interruption protocols that do not limit participation of working adults.

**Methods:**

We used a hybrid Delphi method, involving 4 iterative survey rounds. Initial surveys were open-ended and broad, refining over subsequent rounds into more specific, closed-ended questions based on previous feedback. Between rounds, an independent stakeholder group reviewed interim findings, incorporating a nominal group technique to enhance the process. Panelists represented diverse racial, ethnic, sex, and gender perspectives, including an intentional oversampling of experts on racial and ethnic minority issues. Recruitment was facilitated through partnerships with community-based organizations, such as The Well Project, National Minority AIDS Council, and TruEvolution.

**Results:**

As of December 2024, all 4 Delphi survey rounds and 3 nominal group technique discussions have been completed. The process progressed from broad, open-ended questions in round 1 to structured ranking and rating in rounds 3 and 4. Iterative feedback informed survey refinement between rounds. The final data analysis and synthesis of consensus recommendations are underway and will be reported in a forthcoming results paper.

**Conclusions:**

The hybrid Delphi methodology effectively refined responses and built consensus on engaging priority populations in HIV cure research. Oversampling of diverse participants and the inclusion of independent stakeholder feedback added robustness and inclusivity to the findings. Future steps include detailed data analysis and data dissemination. Consensus recommendations will be reported in subsequent manuscripts to inform more inclusive, trust-centered, and accessible HIV cure trial design.

**International Registered Report Identifier (IRRID):**

DERR1-10.2196/67123

## Introduction

### Background

Several major advancements in the areas of therapeutics for the prevention and treatment of HIV have made finding a cure more possible than impossible [1]. However, as the field of HIV cure research grows in the number of conducted and planned studies, and in the complexity and intensity of potential cure-related strategies, ethical and social challenges emerge that must be addressed [2-4]. Furthermore, several HIV cure–related trials require the interruption of HIV antiretroviral treatment (ART)—a process known as analytical treatment interruption (ATI) [5].

Unfortunately, biomedical advancements for ending the HIV epidemic have been associated with a widening of disparities, as people with HIV who carry the greatest disease burden (eg, Black gay men and transgender women) are underrepresented in clinical trials of these advancements. Moreover, they are not the first to benefit once implementation has rolled out. This was true when a new class of combination ART arrived in 1995 [6], which dramatically increased the likelihood of people with HIV achieving viral suppression, and again in 2012, when pre-exposure prophylaxis for HIV prevention was approved. White men who have sex was men were significantly more likely to use pre-exposure prophylaxis than Black men who have sex was men, although Black men represented the population carrying the highest burden of HIV [7-9].

Accurately representing the disease burden and proportionally enrolling people with diverse racial, ethnic, sexual, and gender identities is a key challenge to ending the HIV epidemic. It is well-documented that past demographic characteristics of trial participants do not reflect the diversity of people with HIV in the United States [10-12]. Four reviews or landscape analyses [10-13] reporting on over 100 HIV cure–related studies found that detailed demographic information of trial participants was severely lacking, and in many cases, could not be extracted. When available, almost all racial, ethnic, sex, or gender minority groups made up <20% of trial participants [10]. Furthermore, data on young adults are also limited, as ATI trial participants tend to be older [14,15].

It is important to explore and address sample differences in HIV cure outcomes between demographic subgroups. For example, according to the US Centers for Disease Control and Prevention, 65% of people with HIV in the United States are Black or African American or Latino [16], and although smaller in total numbers, Black or African American cisgender women and transgender women are severely overburdened compared to their White and cisgender counterparts [16]. Currently, HIV cure trial participant samples do not come close to representing the populations carrying the greatest burden of HIV in the United States [17].

Considering the risks posed by HIV cure research, which often involve monitoring interruptions and pauses of HIV ART—also called ATIs [5], additional barriers to participation are likely structural in nature. Recent federal guidance and research on diversifying clinical trial samples identified potential strategies to overcome barriers that exclude certain groups [18-21], such as documenting demographic information, eliminating inclusion and exclusion criteria that are not scientifically justified, listing enrollment targets (eg, by race and ethnicity, as well as sex and gender), and designing study visit schedules that allow for working adults and caregivers to participate [22]. However, given the historical and ongoing maltreatment of people of color in medical settings [23,24], it is important to acknowledge that hesitance to engage in risky research is compounded by issues related to trust and medical mistrust [25-28] and further exacerbated by biases in biomedical teams screening participants based on assumptions about their willingness to participate.

The prevailing call to action as explained by the phrase “nothing about us without us” [12,29-32] makes clear that advancing HIV cure science without the people who are most impacted by HIV in the United States is a major failing of the scientific community. To prepare for the advent of a cure for HIV, we sought to generate consensus on helping address outstanding ethical and social challenges facing the field of HIV cure research regarding priority populations.

### Objectives

In 2018, a consensus meeting [5] organized by the Ragon Institute of Massachusetts General Hospital, Massachusetts Institute of Technology, and Harvard Medical School led to key recommendations for future HIV cure research design. A second ATI consensus meeting, organized by the Ragon Institute and the US Military HIV Research Program, occurred in Nairobi, Kenya, in May 2024, with a focus on building consensus for implementing ATIs in resource-limited settings (report forthcoming). However, these meetings were led by researchers who discussed and set current priorities using an invitation-only panel format, which may not fully consider the needs and priorities of potential HIV cure research participants. To build on prior efforts, such as the ATI-focused consensus meetings led by the Ragon Institute, which were conducted through invitation-only expert panels, we adopted a hybrid Delphi consensus-building approach. The Delphi method offers a more inclusive, systematic, and iterative process, allowing integration of community perspectives, diverse stakeholder engagement, and structured feedback across multiple rounds. This approach is particularly well-suited for addressing ethically complex issues in HIV cure research that require consensus from both experts and affected communities [33,34].

Here, we describe a protocol to build consensus on three research questions: (1) how to better engage populations in HIV cure research who themselves carry the greatest burden of HIV (ie, racial, ethnic, sex, and gender minority groups); (2) how to enhance trust and diminish mistrust in health care and scientific settings that are associated with lower willingness to participate in HIV cure research; and (3) how to design HIV cure research and ATI protocols that do not limit participation of working adults (Table 1). Notably, we focus this discussion on the United States, given the sample recruitment strategy and the professional connections within the investigators’ social networks.

**Table 1:** Initial unresolved challenges in HIV cure–related research.

Potential challenges	Possible questions to panel
Engaging people with HIV in HIV cure research who have been historically excluded, including Black and African Americans, cisgender women, and sexual and gender minority groups	What are the reasons for the underrepresentation of diverse people with HIV in HIV cure research? What are the strategies we can use to engage diverse people with HIV in HIV cure research?
Enhancing trust and minimizing medical mistrust in research, and accepting or tolerating analytical treatment interruptions	What is medical mistrust to you? What types of trust influence research? How does trust and mistrust affect HIV cure research? Analytical treatment interruptions specifically? Whose responsibility is it to address trust and mistrust in HIV cure research? How would you address it in your specific role?
Meeting the needs of adults with full-time responsibilities	How should normal work schedules inform trial protocol designs? How do we operationalize trial activities that do not require extended absences from work?

## Methods

### Hybrid Delphi Process Study Design

The goal of our hybrid Delphi process was to arrive at consensus around challenges facing HIV cure research participation. On the basis of current published recommendations around Delphi methods [35,36], our study consisted of 4 survey rounds. Each survey contained modules corresponding to each of the 3 challenges listed previously. In this approach, each round of data was rapidly and iteratively analyzed to generate consensus around each challenge. The process repeated until no further refinements or considerations emerged around a challenge. The survey rounds began with open-ended and broad questions, then became narrower and closed-ended, in accordance with previous round responses from the study participants. For example, in round 1, participants provided free-text responses to the question “What barriers stand in the way of recruiting more women into HIV cure trials?.” These free-text or qualitative responses were analyzed using a general thematic analysis approach. In round 2, we asked this same question in the following format: “Out of the top five barriers to women being recruited into HIV cure trials your group identified in the previous round, which is most important to you?.” Respondents moved toward ranking responses in round 3 and rating them in order of importance in round 4, that is, “If we have to prioritize one barrier to recruiting women into HIV cure trials, is it A, B, or C?.” Thus, over the course of this process, a consensus was built.

**Figure 1:**
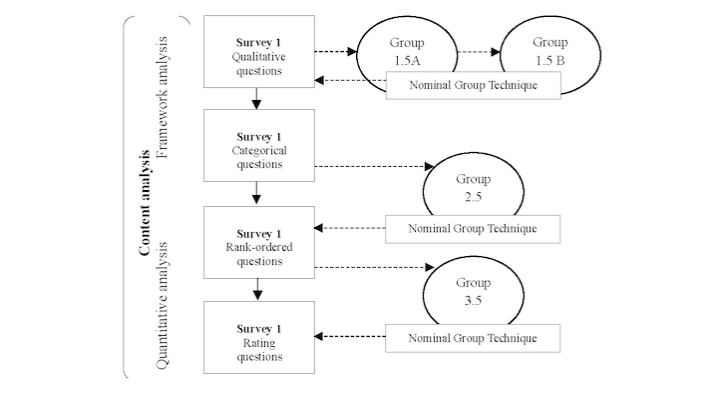
PERSIST Delphi data collection analysis flowchart. NGT: nominal group technique.

To further ensure the robustness of findings, in between the survey rounds 1 and 2, 2 and 3, and 3 and 4, we asked an independent stakeholder group to review the interim survey findings. Implementing this group review introduced a hybrid design [37,38] to the Delphi process, capitalizing on the benefits of a diverse survey response panel and ensuring data validation. In this design, an independent (nonpanel) community expert group comprising 8 participants provided feedback on each survey round in meetings structured according to the nominal group technique (NGT) [35,39]. The NGT minimized “loudest voice” bias possible during the traditional free-flowing focus group format by structuring time for each participant to share their contributions. Then, we aggregated feedback to verify findings and identify information gaps in the emerging data from survey rounds [37,38]. This feedback also informed subsequent survey questions (Figure 1).

The hybrid Delphi design conferred numerous advantages not evident in an invitation-only panel format. The methodology decentralized input, prevented confrontation, increased comfort in raising controversial questions, and allowed for giving controlled feedback in each round [36,40]. Importantly, the technique prevented singular views from dominating or less popular perspectives from being marginalized. The online administration of the survey resolved geographic limitations [36,40] and allowed for confidentiality in response submission. Including multiple expert groups increased representation of professional backgrounds, academic disciplines, and community stakeholder input.

### Eligibility, Identification, and Recruitment of Delphi Panel Members

To meet our objectives, we recruited Delphi panel members (ie, survey respondents) representative of racial, ethnic, sex, and gender minority groups, as well as experts in HIV cure in the United States. Expertise was defined as the ability to represent the viewpoint of each group, based on the number of years in a professional field or in the field of HIV cure research. The panelists, although independent, collectively represented 1 of 6 distinct perspectives: A—community experts and members, B—biomedical researchers, C—HIV care providers, D—funders and private industry members, E—bioethicists, regulators, and institutional review board (IRB) members, and F—individuals studying medical mistrust, medical racism, and community engagement (Table 2). Importantly, to correct for the underrepresentation of racial and ethnic minority groups in HIV cure research, we oversampled the group representing expertise in this topic (group A; projected n of 16 rather than 8). This was done to ensure that perspectives from those most disproportionately affected by HIV were meaningfully reflected in the consensus-building process. The overall sample size was selected to ensure broad input, aligned with previous research [36], and helped preserve management of the panel.

To recruit panel members, we leveraged the community and scientific networks of our investigative team. Specifically, we partnered with 3 highly influential organizations in their respective fields (The Well Project, National Minority AIDS Council, and TruEvolution) to identify key panel members representing diverse groups of people with HIV. The Well Project also assisted with identifying racially and ethnically diverse cisgender women and transgender women who systematically reviewed data and provided feedback during the NGT.

**Table 2:** The initial planned panel composition by the Delphi groups.

Group	Projected, n	Description
A	16	Key persons that represent the *perspectives of people with HIV* across spectrums of age, sex and gender, race and ethnicity, and regions of the United States. Importantly, we oversampled group A to represent the demographic diversity of *people with HIV* (age, race and ethnicity, sex and gender, and US region).
B	8	*Biomedical HIV cure researchers* in the ACTG^a^ network and other HIV cure research groups that have used ATIs^b^ in the past 5 y.
C	8	*HIV primary care providers* (including those not involved in HIV cure research). The inclusion of HIV primary care providers is important to ensure ATIs remain acceptable, and this group has been underrepresented to date.
D	8	*Funder and industry representatives* who provide funding to support HIV cure research.
E	8	*Bioethicists, regulators, and Institutional review board members* who may review HIV cure research.
F	8	*Individuals studying medical mistrust*, medical racism, and community engagement.

^a^ACTG: AIDS Clinical Trials Group.

^b^ATI: analytical treatment interruption.

### Delphi Survey Procedures

In each round, Delphi panelists received, via email: a survey link; an individually assigned 3-character participant ID (PID) number consisting of their respective group designation (A to F) and 2 digits; and a reminder of their respective study group assignment (A to F). Each survey began with video-recorded messages from the investigative team describing the overall study purpose and the round-specific instructions. In round 1, panelists first completed a standard demographic questionnaire, which also included questions about the number of years of professional experience and the number of years worked in HIV cure–related research or health research. Panelists who missed round 1 and entered the study in subsequent rounds completed informed consent and the demographic form as of their entry round. Panelists entered their PID and selected their study group, then proceeded with each survey. Surveys were programmed in Qualtrics XM.

### Contents of Delphi Surveys and Analyses

#### Overview

Demographic data were compiled in SPSS. Data exports from Qualtrics XM were analyzed in Microsoft Excel. In each survey round, we conducted standard quality checks, checking raw data for duplicates, missingness, partial completion, and skip logic, as needed. Responses were both pooled for overall sample analysis and grouped by respondent expertise category (A to F). Spreadsheets were set up such that each row represented a unique response, and each column a unique code.

#### Round 1

In round 1, framework analysis guided our analytic approach [41-43]. Framework analysis is useful when there is a need to organize open-ended responses to prespecified questions over a discrete period. Framework analysis consists of (1) deep familiarization with the data; (2) coding raw data based on *a priori* and emergent themes; (3) finalizing codes in an analytic framework; (4) applying the analytic framework to restructure the data to summarize each narrative; and (5) mapping the data to reduce volume, resulting in a matrix view [41,42].

We adopted an inductive coding approach. Coders (AK and LRG) read the responses, created a code per unique concept within the response, and then recorded this code in a column within a Microsoft Excel sheet. If applicable, they then created subsequent codes in an adjacent column. Once a code was created, the column was dedicated to that code, and the code was listed as the column header. The process continued with subsequent codes. Categories were modified as needed to capture greater breadth. New categories were created to minimize overlap of separate concepts. This procedure resulted in a matrix, with each row corresponding to the unique response, parsed into codes, and each column representing the unique code.

We counted the total number of code occurrences, computing the proportion by dividing the total by the sample size. Next, we obtained an ordered list of responses, from most frequent to least, per question, by first flipping the dataset (via the paste transpose function in Microsoft Excel), then sorting the data in descending order on the percentage column. We then created a 3-part item for each question, categorizing the “most reported,” “less reported,” and “unique” responses. We defined “more,” “less,” and “unique” as follows: (1) more frequent codes (occurring in more than 10% of responses), (2) less frequent codes (occurring in 10% or fewer of the codes), and (3) unique codes (occurring only once). Notably, “consensus” varied, ranging from a low of 10% to 18% in categorized responses to one question, to a high of 10% to 71% in another. We then refined the existing code list. Unique items were further consolidated into “more” or “less” frequently occurring codes, as appropriate. Responses were combined into broader thematic categories and synthesized in a Microsoft Word document.

#### Round 2

On the basis of the round 1 findings, we developed the round 2 survey. We organized the second survey into three main parts: part 1—barriers and burdens to participation in HIV cure research with ATIs, part 2—strategies and solutions to enhance participation in HIV cure research with ATIs, and part 3—general questions and medical mistrust and requiring thresholds in HIV cure research (eg, trials must enroll a minimum percentage of racial and ethnic minority group). In each of parts 1 and 2, the general questions were presented first and the subgroup-specific questions asking about specific populations (ie, Black or African American and Latino populations, cisgender women, and transgender women) followed. In this round, we collapsed the Black or African American and Latino subgroups, given multiple commonalities in respondents’ responses to recognized barriers and proposed solutions within the 2 priority populations.

Respondents were asked a series of bipartite questions. First, select 5 items from the most commonly reported responses; then, select 3 items from the less commonly reported responses. Response items were grouped into topical themes and narrowed down as follows: overall barriers and solutions (Topic 2 from round 1)—8 response items per question and subgroup-specific barriers and solutions (Topic 1 from round 1)—approximately 5 items (notable exceptions: 6 more reported barriers for cisgender women and 4 less reported barriers for transgender women). We rephrased response items for clarity, coherence, and internal consistency, based on reviewer comments.

Once the survey fielding was completed, we analyzed the data. As in round 1, we first conducted standard quality checks. In Microsoft Excel, we sorted and grouped responses by respondent expertise group (A to F). We then calculated raw counts and proportions of responses per response category within each question. This was performed separately for each respondent group (A to F). We then created an overall results display, mapping each response category per question against the quantity and percentage of respondents selecting the relevant response category. Subsequently, we created group-specific results sheets, sorted in descending order. We then conducted a series of data checks, ensuring accuracy in sorting and calculations. Finally, we created bar graphs to visualize the top 5 response categories per question item for each respondent group.

#### Round 3

To maintain parallel construction with the round 2 survey, we organized the round 3 survey into three parts: part 1—barriers and burdens; part 2—strategies and solutions; and part 3—general, medical mistrust and participation thresholds. In parts 1 and 2, we presented the general questions first, and the subgroup-specific questions (Black or African American and Latino populations, cisgender women, and transgender women) followed. As described in the previous section, the presented response choices were those selected by survey respondents in round 2 and analyzed for the third round. The response item wording also paralleled round 2, modified only according to the round 2.5 discussion (refer to *Hybrid Group Discussion* section).

In Qualtrics XM, panelists were first presented with a randomized response list, per question item, of each subgroup choice (A to F). To facilitate comparison across subgroups, each item was color-coded and displayed as a column in a table, per respondent group. Participants received information summarizing their group’s responses in comparison with those of other groups. To generate consensus, they were instructed to consider the group-specific results before proceeding to ranking. They were then instructed to rank the pooled responses in order of descending importance (from most to least important). Participants ranked 5 answer choices for 2 questions on general barriers and solutions and 3 answer choices each for subgroup-specific questions.

We analyzed round 3 data in Microsoft Excel, first conducting quality checks, and then performing calculations. Per the branching in this round of the Qualtrics XM survey, this process consisted of first analyzing 6 separate datasets, one per group. For each, we calculated the mean value of each item’s rank, per group (A to F). Given the need to recategorize participants who misclassified themselves into an incorrect group, this was done using stacked ranges. Once calculated, we then created separate worksheets per question item (Q1-Q10). Within each, we converted the average values back into ranks. We then displayed each group’s rankings of each response item, from first to last place. In one case, where only miscategorized (and then reassigned) participants ranked a specific response option, we used counts, rather than averages, to determine rankings. Finally, we created stacked bar charts of the counts of the number of groups selecting each answer choice, from first to last place, for each question item.

#### Round 4

We organized the final survey into three parts, consistent with the previous round: part 1—barriers and burdens, part 2—strategies and solutions, and part 3—general. We asked the overall population questions first, followed by subgroup-specific questions (Black or African American and Latino populations, cisgender women, and transgender women). Participants also completed an open-ended summary section (refer to *Delphi Survey Progression* section).

In Qualtrics XM, participants viewed previous round survey results in 2 graphics per question: a color-coded bar graph indicating *how many* groups ranked each response choice, from first to last, and a data table displaying *how each group* (A to F) ranked each response choice, from first to last place. In part 1, participants were asked to rate, on a Likert scale, “how important do you believe each barrier is to address on a scale from 1 (equal importance to other items) to 9 (extremely important (highest priority)).” In part 2, they were asked to rate, on a Likert scale, “how urgent do you believe each solution is to address on a scale from 1 (equal relative urgency) to 9 (extremely urgent [highest priority]).” Scales were presented for each response item within each question.

Participants thus rated 5 answer choices for each of the 2 questions on general barriers and solutions, and 3 answer choices for each of the 6 subgroup-specific questions. Notably, per the prevalence of missing data and several tied responses, we dropped supplementary part 2 questions (Q5a, Q6a, Q7a, and Q8a), which asked participants to rate less frequently recommended solutions in the previous round.

As in previous iterations, we analyzed participant responses, first conducting quality checks, then calculations. After verifying existing PIDs on file, we sorted the dataset by respondent group (A to F), then calculated means and SDs for each numeric-response question item (Q1-Q8) response ratings. In Q1 and Q5, respondents rated 5 responses; in all other questions (Q2-Q4 and Q6-Q8), they rated 3 responses. We then sorted the transposed response lists to obtain a descending ratings list for each item. We performed these operations for the pooled response set and for each subgroup (A to F). We also highlighted the occurrences of tied ratings.

Next, we created combination line and bar charts for each barrier and solution category: overall participation, Black or African American and Latino populations, cisgender women, and transgender women. We represented each group’s mean ratings via bar charts and the pooled responses as an average line, within each chart, thus showing both group-specific ratings and overall trends. Separately, we processed the open-ended responses (Q9-Q11), the latter of which is presented here (refer to *Results* section and Multimedia Appendix 1).

### NGT: Hybrid Group Discussions

Between each survey round, Delphi panel results were reviewed by an independent group discussion with members referred by The Well Project, an HIV community organization focused on the needs of women, members of which represent the diversity of women living with or affected by HIV in the United States (Figure 1). The discussions were held via teleconference and moderated by a Well Project–affiliated consultant (BP). Participants filled out demographic information and reviewed the study information sheets. As a group, NGT participants reviewed a Microsoft PowerPoint presentation highlighting key survey findings from the previous survey round.

The in-between round 1 and round 2 discussion (round 1.5) centered on general participation issues specific to HIV cure research and ATIs, as well as issues specific to 2 populations of interest: cisgender women and transgender women. This discussion was split into 2 parts, each about 90 minutes in length. Seven participants were involved in the first part of the round 1.5 discussions, 6 of whom also participated in the second part. In round 1.5, participants were asked to review findings on barriers and solution to participation in HIV cure research, general and specific to women (cisgender women and transgender women), then select their top 3 to 4 choices. They also identified gaps in round 1 survey results—for example, discussion around “transphobia” (fear) as a barrier to participation expanded to include “transmissia” (hostility). Items emphasized during the in-between group discussion were marked with asterisks (*) in the round 2 survey.

The in-between round 2 and round 3 discussion (round 2.5) consisted of participants ranking their top 3 or 4 choices for top strategies to better engage cisgender women and transgender women in HIV cure research. Six participants reviewed responses from each of the survey respondent groups (A to F) during the 90-minute discussion. While overlap and variation existed, participants generally emphasized centering the study design on each group’s specific needs, including prioritizing the meaningful involvement of people with HIV [44]. Insights from the hybrid group discussion led to rephrasing of 3 survey items (overall, cisgender women, and transgender women) to include the word “unique” in the round 3 survey.

Eight participants attended the final, hour-long, in-between round 3 and round 4 discussion (round 3.5). Participants first anonymously ranked strategies for engaging cisgender and transgender women in HIV cure research, then discussed overall and group-specific findings. Participants discussed the practical implementation of “true inclusion” for transgender women and the intersectional identities of cisgender women. They also shared feedback regarding the Delphi consensus-building process and the NGT, noting challenges in ranking and appreciation for a safe space to discuss transgender and cisgender identity.

### Ethical Considerations

The hybrid Delphi consensus-building process was approved by the University of California, San Diego IRB (project #805348). All participants provided electronic informed consent before enrollment. Participation was voluntary, and panelists or NGT participants were informed of their right to decline or withdraw at any time without penalty. Data collected during the Delphi rounds and NGT discussions were deidentified before analysis to ensure participant confidentiality. No personally identifying information was retained. Participants received US $100 compensation per completed survey round, and an additional US $100 bonus for completing all 4 rounds, for a maximum of US $500 total. NGT participants were also compensated US $100 per discussion (US $200 for round 1.5 if they participated in both sessions). The funders and government officials who participated were not compensated. These procedures were approved by the IRB as part of the study protocol. To support transparency and completeness in reporting, this protocol has been mapped to the DELPHISTAR (Delphi studies in social and health sciences—recommendations for an interdisciplinary standardized reporting) reporting guidelines for Delphi studies [45].

### Recruitment and Enrollment of Delphi Panel Members

Potential panelists received a link to our study website, which provided an overview of the Delphi process. The invitation materials explained the study purpose and expected time commitment (30-60 min per survey round). Panel recruitment lasted 7 weeks, from February 27, 2023, to April 14, 2023. The round 1 survey data collection lasted 4 weeks, from April 10, 2023, to May 9, 2023. Round 2 data collection lasted 6 weeks, from September 15, 2023, to October 20, 2023. Round 3 data collection lasted 6.5 weeks, from March 19, 2024, to May 3, 2024. Round 4 data collection also lasted 6.5 weeks, from July 10, 2024, to August 23, 2024.

We initially invited 85 potential panelists to participate in the Delphi consensus-building panel (Table 3). Of these 85, 58 (68%) accepted the invitation. One panelist later withdrew due to retirement. Of the remaining 57, 52 (91%) completed the round 1 survey. In round 2, 82% (46/56) completed the survey. In rounds 3 and 4, 86% (48/56) completed the survey (Table 4).

**Table 3:** Delphi panel enrollment (United States, 2023 to 2024).

Group	Group name	Invited (N=85), n (%)	Accepted (n=58), n (%)	Declined (n=12), n (%)	No response (n=14), n (%)	Accepted and withdrew (n=1), n (%)
A	Community experts and members	20 (24)	17 (29)	0 (0)	3 (20)	0 (0)
B	Biomedical researchers	13 (15)	9 (15)	2 (17)	2 (13)	0 (0)
C	HIV care providers	11 (13)	9 (15)	1 (8)	1 (7)	0 (0)
D	Funders and private industry members	14 (16)	7 (12)	4 (33)	3 (20)	1 (100)
E	Bioethicists, regulators, IRB^a^ members	15 (18)	8 (14)	4 (33)	3 (20)	0 (0)
F	Individuals studying medical mistrust, medical racism, and community engagement	12 (14)	8 (14)	1 (8)	2 (13)	0 (0)

^a^IRB: institutional review board.

**Table 4:** Number of invited and completed panelist surveys.

Group	Round 1				Round 2				Round 3				Round 4		
	Invited (n=57), n (%)	Completed (n=52), n (%)	Completion rate (%)	Invited (n=56), n (%)		Completed (n=46), n (%)	Completion rate (%)	Invited (n=56), n (%)		Completed (n=48), n (%)	Completion rate (%)	Invited (n=56), n (%)		Completed (n=48), n (%)	Completion rate (%)
A	17 (30)	17 (33)	100	17 (30)		16 (35)	94	17 (30)		15 (31)	88	17 (30)		15 (31)	88
B	9 (16)	8 (15)	89	9 (16)		5 (11)	56	9 (16)		6 (13)	67	9 (16)		5 (10)	56
C	9 (16)	9 (17)	100	9 (16)		9 (20)	100	9 (16)		9 (19)	100	9 (16)		9 (19)	100
D	6 (11)	4 (8)	67	5 (9)		4 (9)	80	5 (9)		4 (8)	80	5 (9)		4 (8)	80
E	8 (14)	7 (13)	100	8 (14)		6 (13)	75	8 (14)		7 (15)	88	8 (14)		8 (17)	100
F	8 (14)	7 (13)	88	8 (14)		6 (13)	75	8 (14)		7 (15)	88	8 (14)		7 (15)	88

### Delphi Panel Demographics

**Figure 2:**
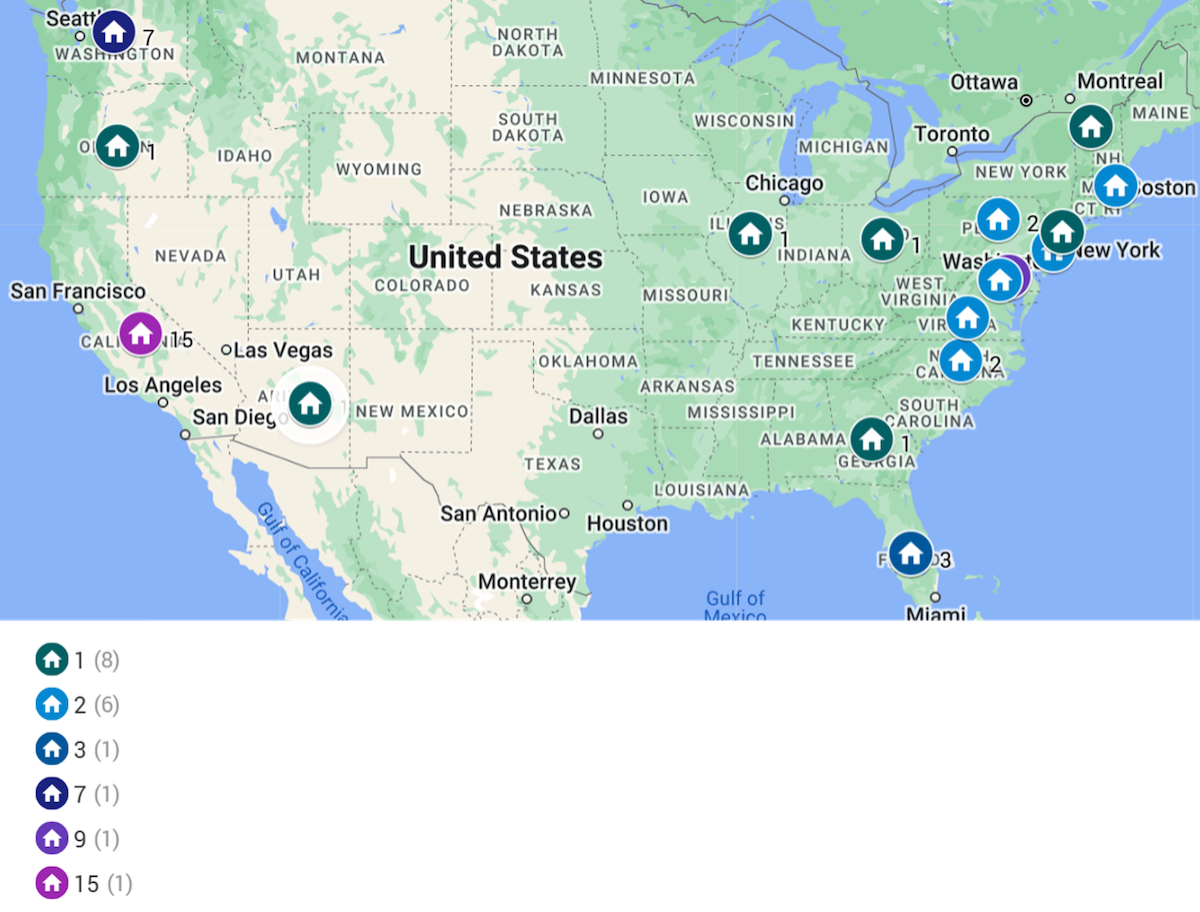
Map of the Delphi participants across the United States (2023 to 2024).

For descriptive purposes, panelist demographic details are shown in Table 5. The panelists were generally concentrated in the west or northeast regions of the United States (Figure 2).

**Table 5:** Demographic characteristics of the Delphi panel (United States, 2023 to 2024; n=54).

Characteristic			Panelists, n (%)
**Gender identity**			
	Cisgender woman	34 (63)	
	Transgender woman	2 (4)	
	Cisgender man	15 (28)	
	Nonbinary or gender queer	2 (4)	
	Unknown	1 (2)	
**Sex assigned at birth**			
	Female	35 (65)	
	Male	19 (35)	
**Ethnicity**			
	Hispanic or Latino	7 (13)	
	Not Hispanic or Latino	46 (85)	
	Unknown	1 (2)	
**Race**			
	Asian	6 (11)	
	Black or African American	11 (20)	
	Mixed or other	3 (6)	
	Prefer not to answer	1 (2)	
	Unknown	1 (2)	
	White	32 (59)	
**Education level**			
	High school diploma or General Educational Development	1 (2)	
	College graduate	10 (19)	
	Master or equivalent	8 (15)	
	Doctorate or equivalent	35 (65)	
**US residence region**			
	Northeast	19 (35)	
	Southeast	8 (15)	
	Southwest	1 (2)	
	Midwest	2 (4)	
	West	23 (43)	
	Prefer not to answer	1 (2)	
**Number of years worked in the field (HIV research: 19.7 y)**			
	1 to 10	8 (15)	
	11 to 20	21 (39)	
	21 to 30	17 (31)	
	31 to 40	5 (9)	
	>41	2 (4)	
	Prefer not to answer	1 (2)	
**Number of years in HIV cure–related research (HIV cure research 7.9 y)**			
	0	9 (17)	
	1 to 5	13 (24)	
	6 to 10	19 (35)	
	11 to 15	4 (7)	
	16 to 20	2 (4)	
	>21	4 (7)	
	Prefer not to answer	2 (4)	
	Unknown	1 (2)	

### NGT or Hybrid Group Participant Demographics

Table 6 also lists the demographics of 8 participants who participated in the group discussions referred to by The Well Project.

**Table 6:** Nominal group technique participants (n=8)—demographic characteristics (United States, 2023 to 2024).

Characteristic		Participants, n (%)
**Gender identity**		
	Cisgender woman	6 (75)
	Transgender woman	2 (25)
**Sex assigned at birth**		
	Female	6 (75)
	Male	2 (25)
**Ethnicity**		
	Hispanic or Latino	1 (13)
	Not Hispanic or Latino	7 (88)
**Race**		
	Black	6 (75)
	White	2 (25)
**Highest level of education**		
	High school diploma or General Educational Development	1 (13)
	Some college	4 (50)
	College or above	2 (25)
	Prefer not to answer	1 (13)
**US region**		
	West	1 (13)
	Southwest	2 (25)
	Midwest	1 (13)
	Southeast	1 (13)
	Northeast	3 (38)
**Occupation or field of work**		
	Public health	1 (13)
	Cashier	1 (13)
	Peer navigator	1 (13)
	HIV and trans advocacy	3 (38)
	HIV clinical work	2 (25)
**Years involved or worked in the field of HIV**		
	0 to 5	3 (38)
	6 to 10	1 (13)
	11 to 15	2 (25)
	16 to 20	2 (25)
**Familiarity with the field of research**		
	Not at all familiar	1 (13)
	Somewhat familiar	3 (38)
	Moderately familiar	2 (25)
	Very familiar	2 (25)

## Results

### Delphi Survey Progression

The round 1 survey consisted of 35 free-response questions, organized in 3 topics: engaging underrepresented populations (20), general participation barriers and strategies (16), and medical mistrust (9). Within the first topic, 4 questions (reasons for lack of representation, barriers, recommendations for researchers, and strategies) were repeated for each of the 4 groups: Black or African American individuals, Latino individuals, cisgender women, and transgender women. For each topic, participants were also asked to consider solutions without restrictions on resources, as well as barriers to such solutions (Textbox 1).

**Delphi survey modules, by survey round, with the number of questions.**

**Round 1 (35 questions)**

- Engaging marginalized groups (20 questions)
- Engaging all groups (16 questions)
- Medical mistrust (9 questions)

**Round 2 (17 questions)**

- Barriers and burdens (8 questions)
- Solutions and strategies (8 questions)
- Medical mistrust and required thresholds (3 questions)

**Round 3 (14 questions)**

- Barriers and burdens (4 questions)
- Solutions and strategies (8 questions)
- Required thresholds and summary (4 questions)

**Round 4 (31 questions)**

- Barriers and burdens (14 questions)
- Solutions and strategies (14 questions)
- Required thresholds and summary (3 questions)

In round 2, we consolidated items, halving the number of survey questions (to 17), and changing the question type, such that participants were asked to select from listed categories. We also amended the survey structure: rather than grouping by the original topics, we first presented all questions on barriers (generally and then for underrepresented groups), then on strategies, and finally on a summary section (views on required thresholds—yes, no, maybe, and why; and medical mistrust). As stated earlier, because responses indicated that the 2 groups faced very similar barriers to participation, we collapsed the Black or African American and Latino groups into one; we kept questions on cisgender women and transgender women separate, because responses indicated differences in barriers.

In round 3 (16 questions), we maintained the survey structure from round 2 and asked the participants to rank order the items they previously selected in round 2. In topic 1, we focused only on the more frequently reported barriers. In topic 2, we included supplemental questions for less frequently reported solutions. In topic 3, we expanded the question on required thresholds into a 3-part item, which included a “yes, no, and maybe” response, a free-text response, and a select one item. As noted, while participants were asked to rank the pooled survey results, they were also presented with group-specific response choices.

While we maintained the 3-part survey structure, we made notable revisions in the final survey round, resulting in 31 questions. In both part 1 (barriers and burden) and part 2 (solutions and strategies), we asked participants to assess the relative importance (Q1-Q4) and relative urgency (Q5-Q8) of each survey response item. We asked the participants to indicate their ratings using a 9-point Likert scale, where 1=equal relative importance or urgency and 9=extremely important or urgent (highest priority). Thus, each section was expanded to include 14 questions. Finally, we revised the summary section to include only open-ended questions. Having obtained sufficient data in round 3, we dropped the medical mistrust question. The required threshold question was only asked of respondents who had answered “maybe” or “no” to threshold use in the previous round. All participants were asked the 2 remaining summary questions.

### Survey Panel Feedback

As we concluded our last survey round, we asked participants about their experiences as Delphi panelists. A total of 23 participants responded, mostly expressing positive feedback, although 5 highlighted potential areas of improvement (Multimedia Appendix 1). Participants noted that the Delphi consensus-building process was well organized and thought out. The participants were grateful to have been part of a study that valued community input and engagement. Many expressed hope that the study results would lead toward advancements in health equity and greater representation of underserved communities, not only in HIV cure–related research, but also in other fields. In reference to critiques, participants shared that the survey duration was quite long, especially in the first 2 rounds. Not only did survey completion seemed longer than anticipated, but some participants were also unaware that one could pause the survey and resume it at a later time. This caused some discontent among those who wanted to answer more thoroughly but felt constrained due to time limitations. Participants also noted some difficulties with having to rate strategies. At times, participants selected multiple strategies as “urgent”—instead of prioritizing one or the other—due to perceived equal importance.

### NGT Implementation

In the first NGT meeting, participants openly vocalized their responses, which could have influenced others’ responses. To address this, in the second discussion group, the facilitator instructed participants to type in their responses in the Zoom (Zoom Communications, Inc) chat to provide unbiased opinions before engaging in dialogue. In the final discussion group, they were asked to send their chat responses in private messages before discussing openly. Furthermore, our team facilitated a debrief session after the final NGT group. Overall, participants expressed satisfaction, stating how comfortable and safe they felt in sharing their responses. Participants emphasized the importance of continuing conversations around cisgender and transgender women’s health; appreciated learning from one another; and valued how unifying and nondivisive the conversation felt. However, participants found it somewhat difficult to rank their top strategies and burdens. They shared feelings that solutions needed to occur as a systemic cascade, rather than highlighting just 1 or 2 strategic recommendations. Similarly, participants noted that impediments to participation may not be limited to 1 or 2 barriers; rather, the intersection between individual and systemic level barriers needed to be addressed.

## Discussion

### Anticipated Findings

This protocol is designed to yield consensus-driven recommendations informed by both experts and community perspectives on 3 persistent challenges in HIV cure–related research. We anticipate that the findings will offer practical, community-validated strategies to improve equity in trial design, particularly by clarifying how representation, trust, and trial accessibility can be addressed in ATI-inclusive protocols. Given the hybrid Delphi-NGT structure, we also expect insights on how participatory approaches can strengthen consensus-building efforts in ethically complex research domains. This methodology may be transferable to other fields, including oncology, reproductive health, or vaccine research, where inclusion, community engagement, and ethical complexity are similarly critical.

### Advantages and Disadvantages of Hybrid Delphi Methodology

The Delphi methodology used in this study conferred several significant advantages. The iterative nature of the Delphi process, involving 4 rounds of surveys, allowed a systematic refinement of responses and a progressive convergence toward consensus around the topic of prioritizing key strategies to engage priority populations in HIV cure research. Initially, broad and open-ended questions facilitated a comprehensive exploration of challenges around the topic of barriers and then solutions to engage populations in this research, which were subsequently distilled into more focused queries as the Delphi consensus-building process progressed [38]. This approach ensured that the resultant consensus was grounded in a thorough and nuanced understanding of the issues surrounding HIV cure research participation. In addition, the recruitment strategy aimed to enhance the study’s inclusivity and representativeness. By engaging a diverse panel of stakeholders, including members from underrepresented racial and ethnic groups, community experts, biomedical researchers, and HIV care providers, the study collected a wide range of perspectives. The intentional oversampling of racially and ethnically diverse participants further addressed critical representation gaps, ensuring that the findings reflected a broad spectrum of experiences and viewpoints. Finally, the hybrid design, incorporating feedback from an independent stakeholder group through the NGT, added robustness to the Delphi consensus-building process. This design allowed for the validation of data and the introduction of new insights, thereby mitigating potential biases and enhancing the reliability of the results. The structured nature of the NGT discussions also minimized the risk of dominant voices overshadowing less popular but important viewpoints, fostering a more balanced and comprehensive analysis [35].

Despite these advantages, several limitations of the Delphi methodology were evident. The reliance on online surveys, while facilitating wide geographical and disciplinary participation, may have introduced accessibility barriers for potential respondents, potentially limiting the diversity of input. The initial Delphi survey round was open-ended, and thus more onerous and time-consuming than the subsequent ones. In addition, the iterative rounds of the Delphi process, while beneficial for refining consensus, could have led to survey fatigue among participants, potentially affecting response quality and engagement over time. Another limitation was the potential for bias in the selection and recruitment of panel members. While efforts were made to ensure a diverse and representative panel, the initial sample size and recruitment strategy could have inadvertently excluded some key stakeholders. For instance, the predominance of participants from the west and northeast regions of the United States might not fully capture the perspectives of those from other geographic areas. Furthermore, the high level of education and professional experience among participants may not fully reflect the broader population’s views, potentially skewing the findings toward more informed or specialized perspectives. Finally, the Delphi process’ complexity and multiple feedback rounds presented challenges in data interpretation and synthesis [46]. The iterative surveys, while enabling a thorough exploration of perspectives on HIV cure research participation, also introduced variability in responses, complicating the interpretation and the consistency of consensus signals.

### Lessons Learned

In categorizing the survey panel respondent groupings (eg, biomedical researchers vs bioethicists), we learned that the assigned categories were more porous than originally anticipated. As detailed throughout this paper, we divided the sample into 6 groups based on expertise. However, in the third survey round, 3 respondents whom we had categorized as medical mistrust experts (group F) self-identified as community experts and members (group A) and 2 HIV care providers (group C) self-identified as biomedical researchers (group B). While we corrected this by reassigning group membership in the analysis and restructuring subsequent round identification questions, we learned that additional communication with study participants around group membership designation was needed. Moreover, future studies can consider alternative approaches to panel member classification, including asking potential participants to self-identify.

Throughout the data collection period, we allowed multiple survey log-in attempts. In the initial survey round, incomplete entries totaled about a fifth of overall survey attempts; in successive rounds, these dropped to 1 or 2 cases per round. The change in survey format, from primarily open-ended to primarily close-ended questions, likely correlated with initial attempt completion. However, it is worthwhile to consider a single survey log-in to facilitate data processing. This would also serve to eliminate duplicate response entries, although these were not highly prevalent (4 cases in the initial round to 1 or 2 in ensuing surveys). Notably, we recommend communicating with participants regarding the need to save their progress and returning to complete before survey submission.

Previous work, on which our study was primarily modeled [37,38], incorporated “subsequent reduction” of content following initial round open-ended responses. However, in our study an expansion (in round 4) followed the reduction (in rounds 2 and 3). This was due to the change from a ranking of response options in relation to one another to a rating of each individual choice [47]. We did not find this expansion to increase respondent burden. In fact, the round 4 respondent feedback, describing the later rounds of the survey process, was generally positive. Earlier rounds were seen as more time-consuming than expected, which would need to be communicated better in future Delphi studies.

### Retention

Our implementation of the Delphi hybrid methodology proved to be an effective means of participant retention over time. Despite some fluctuations, >80% of eligible participants completed each survey round. On the basis of the participant feedback, we can infer that the decrease in participant burden, as surveys shifted from primarily open-ended to primarily close-ended, facilitated renewed participant commitment. Notably, the final completion incentive for those who engaged in all 4 rounds may have also encouraged retention. The participants also commented that the survey reorganization—from subgroup-specific to orientation around challenges and solutions—allowed for simpler navigation. It is likely that the modality—online access through a popular survey application (Qualtrics XM)—also contributed to participant retention [37]. Similarly, survey respondents’ social ties to the investigative team and existing expertise in the topic matter may have also motivated intrinsic interest in survey completion.

### Limitations

We acknowledge several limitations of our consensus-building process. First, recruitment through investigative team networks may have introduced sampling bias and limited the representativeness of the panel. While participant lists were reviewed by the study team to maximize diversity, this convenience sampling approach may affect reproducibility. Future studies could consider broader outreach or randomized strategies, although these may be more resource-intensive. Second, despite our efforts to ensure demographic diversity, like other hybrid Delphi studies [37,38], the panel was skewed toward highly educated, White, and female participants, with geographic clustering in the northeastern and western regions of the United States. Oversampling was used to address some gaps, but more structured quotas may help ensure even representation in future studies. Third, participant expertise levels varied, and some individuals reclassified their group affiliations midstudy. We corrected for this in later rounds, but future protocols may benefit from allowing participants to self-identify expertise from the outset. Fourth, although our 68% acceptance rate and our total sample size match what is usually expected in Delphi studies (around 60% to 80%), there is still a chance that people dropping out could affect the results. To help prevent this, we used strategies such as offering incentives, making the surveys easier to complete, and staying in direct contact with participants.

### Conclusions

This protocol engaged 6 stakeholder groups to identify barriers to, and strategies for improving, the inclusion of underrepresented demographic groups in HIV cure–related research in the United States. The hybrid Delphi approach, integrating 4 rounds of structured surveys with NGT discussions, proved to be an effective and inclusive method for building consensus across diverse perspectives. Final analysis of consensus findings is underway and will be presented in a separate manuscript. These recommendations will also inform policy briefs, community reports, and presentations at HIV cure research forums. Our team is committed to disseminating these recommendations through academic and community channels to help inform more inclusive HIV cure trial design and implementation.

## Appendix

Multimedia Appendix 1 Supplementary tables.

## Abbreviations

ART: antiretroviral treatment
ATI: analytical treatment interruption
IRB: institutional review board
NGT: nominal group technique
PID: participant ID

## Data Availability

Deidentified data, interview guide, and survey instruments used in this study are available upon reasonable request from the corresponding author.
