# Supplementary material for: Resolving Challenges in HIV Cure–Related Research: Protocol for a Modified Delphi Consensus-Building Process
Source: JMIR Res Protoc. 2025 Aug 6;14:e67123. doi: 10.2196/67123 (PMC12368466; doi:10.2196/67123)
Supplement: Multimedia Appendix 1 [file resprot_v14i1e67123_app1.docx]

| **Supplementary Table 1: Delphi Consensus-building Process Feedback** | | |
| --- | --- | --- |
| **Themes** | **Subthemes** | **Exemplary Quotations** |
| **Positive Aspects** | Pleased or honored by the opportunity to participate | *It has been a pleasure to participate in this very important endeavor and I look forward to next steps and great outcomes.*  *It has been an honor to be invited to participate in this. I hope more studies seek out community input in the way you have. It seems like a novel approach, and such an important one for research like this. Thank you!* |
|  | Gratitude towards research and community input | *This research you are doing is very important for our field, many of us have been working on and hoping for effective functional HIV cure for decades. Now that the science is advancing, we need to address health equity and participation representation in all our studies, no matter how challenging. I applaud all your efforts and appreciate the opportunity to contribute my thoughts and experience to this project.*  *This has been very encouraging and inspiring to provide a community voice and share concerns about barriers, support, inclusion... this is so important, and we cannot wait for results and recommendations because there can apply to other research.*  *I want to thank you all for the work that you were doing for us people living with HIV and those that work in the research for the cure. So we could all be free one day from this human condition.* |
|  | Impressed by the study team’s efforts [great study process, organization] | *I was very impressed by the thoroughness and thoughtfulness of each survey. It was readily apparent how much work and thought had gone into each one. Thank you very much for including me in this important work.*  *This has been a much better process for participation and could be a model for future engagement. I found it to be manageable and the incentives were fair and effective. I agree that the earlier rounds required more time than indicated. I hope that we get to hear the outcome of the research in some forum, and I sincerely hope that it leads to change. Thanks for the opportunity to participate*  *I have absolutely loved this study! It was so well organized! I felt appreciated and my input was valued. For that reason, I would always sit down and give my full attention and effort on these study questions. So, thank you!* |
| **Areas of Improvement** | Difficulty rating strategies [due to equal importance of multiple options and/or uncertainty while rating responses] | *This last survey I hope I did it correctly, but I read [rated] it a few things nine because I found it to be extremely urgent. Sometimes one of the options was not enough.*  *I was not 100% clear as to whether I could assign the same number to different responses in the same category. I tried to only give one "1" and one "9” but wasn't sure if I could give two "8" responses in a section.* |
|  | Survey display concerns | *Some of the previous surveys were long and winding. I was not sure how you make heads or tails out of responses. There still does not seem to be a definite way to pause the survey and come back to it.* |
|  | Study duration longer than anticipated [especially for earlier rounds] | *These things always take longer than anticipated for people who really try to provide comprehensive thoughtful answers.*  *It was hard to participate during round one, when it felt like I was trapped in an endless series of similar questions, forced to see the survey threw. Once I learned that I could save answers, or step away, the other remaining rounds were much easier to engage with. I hope this work changes our current trajectories for HIV cure-related research and creates more opportunities for community education and inclusion.* |
